# Photoactivation of a Mechanosensitive Channel

**DOI:** 10.3389/fmolb.2022.905306

**Published:** 2022-06-28

**Authors:** Fucsia Crea, Antreas Vorkas, Aoife Redlich, Rubén Cruz, Chaowei Shi, Dirk Trauner, Adam Lange, Ramona Schlesinger, Joachim Heberle

**Affiliations:** ^1^ Department of Physics, Experimental Molecular Biophysics, Freie Universität Berlin, Berlin, Germany; ^2^ Department of Physics, Genetic Biophysics, Freie Universität Berlin, Berlin, Germany; ^3^ Leibniz-Forschungsinstitut für Molekulare Pharmakologie (FMP), Berlin, Germany; ^4^ Department of Chemistry, New York University, New York, NY, United States

**Keywords:** MscL, photolipids, AzoPC, nanodiscs, FTIR spectroscopy, Langmuir film, biomembrane, electrophysiology

## Abstract

Optogenetics in the conventional sense, i.e. the use of engineered proteins that gain their light sensitivity from naturally abundant chromophores, represents an exciting means to trigger and control biological activity by light. As an alternate approach, photopharmacology controls biological activity with the help of synthetic photoswitches. Here, we used an azobenzene-derived lipid analogue to optically activate the transmembrane mechanosensitive channel MscL which responds to changes in the lateral pressure of the lipid bilayer. In this work, MscL has been reconstituted in nanodiscs, which provide a native-like environment to the protein and a physical constraint to membrane expansion. We characterized this photomechanical system by FTIR spectroscopy and assigned the vibrational bands of the light-induced FTIR difference spectra of the *trans* and *cis* states of the azobenzene photolipid by DFT calculations. Differences in the amide I range indicated reversible conformational changes in MscL as a direct consequence of light switching. With the mediation of nanodiscs, we inserted the transmembrane protein in a free standing photoswitchable lipid bilayer, where electrophysiological recordings confirmed that the ion channel could be set to one of its sub-conducting states upon light illumination. In conclusion, a novel approach is presented to photoactivate and control cellular processes as complex and intricate as gravitropism and turgor sensing in plants, contractility of the heart, as well as sensing pain, hearing, and touch in animals.

## Introduction

Mechanosensitive proteins are recognized as important physiological sensors which support the function of vital biophysical systems and facilitate their sensitivity to nearby physical changes (Hunte and Richers, 2008; Eyckmans et al., 2011). A sub-class of mechanosensitive proteins does not require a complex cascade of molecular events to mediate gating, but is activated by just membrane tension. The simplicity of these proteins makes them a useful addition to our bioengineering toolbox. The relationship between mechanosensitive proteins and the lipid membrane is fundamental to their sensing capabilities (Perozo, 2006). The mechanosensitive channel of large conductance (MscL) is an established model system of mechanosensitivity and provides a benchmark for signal transduction within tension-gated proteins (Kung, 2005; Bavi et al., 2017a). In fact, the force-from-lipid theory of mechanosensitive gating was born from the early investigation of MscL; changes in lateral tension in the surrounding bilayer mimic cell turgor and pull the transmembrane helices of the protein, causing the channel to open its pore (Martinac et al., 1990; Moe and Blount, 2005; Jeon and Voth, 2008; Teng et al., 2015). The protein has several substates during its reconformation from closed to open, each of which have an increasing conductance (Sukharev et al., 1999). The N-terminal helix, located at the interface of the solvent and bilayer, is an integral sensor, which when pulled by forces in the bilayer, tilts its neighboring transmembrane-1 helix to trigger an intermediate state of pore opening (Perozo et al., 2002; Bavi et al., 2016). Furthermore, the interface of the protein and lipid bilayer is a significant component of channel regulation; the hydrophobic mismatch caused by changing the bilayer thickness can significantly alter the open probability, as the protein changes its structure in the membrane to conserve its hydrophobic domain within the bilayer (Perozo et al., 2002; Nomura et al., 2012).

Bilayers of 4-*azo*-5P, a photoswitchable lipid mimic, have been shown to facilitate and modulate the folding of non-functional, monomeric MscL into its final, functional, homopentameric state in the membrane. Here, it was speculated that the photoswitching lipid changes the lateral tension in the headgroup region of the bilayer, allowing the protein to easily insert and orient itself in the bilayer (Miller et al., 2016). The same photoswitchable pseudo-lipid was used for the first proof of light-triggered mechanical activation of MscL (Folgering et al., 2004). In a patch-clamp experiment, Folgering et al. proved that they could reversibly increase and decrease MscL activity, when reconstituted in a membrane containing 20% of 4-Azo-5P, by respectively light-switching the lipid mimic from *trans* to *cis* and *vice versa*, for at least five cycles. They speculated that the difference in mass distribution between the more packed trans state and the more disordered cis state of 4-Azo-5P would influence the energetic barrier that needs to be overcome to stretch the membrane.

The photoactive molecule used by Folgering et al., 4-Azo-5P, although being amphiphilic, is actually more a detergent-like molecule rather than a glycerophospholipid*.* Here, we apply AzoPC, a commercially available phospholipid with an azobenzene moiety incorporated into the *sn-*2 lipid tail. It is a reversible photoswitch, which when illuminated with UV light, will isomerize to form its *trans* to the *cis* state. *Trans* AzoPC undergoes isomerization to the *cis* state upon illumination with blue light, or through thermal isomerization which, however, takes a few hours (Pernpeintner et al., 2017). When AzoPC is photoswitched, the membrane undergoes a change in tension profile, bilayer fluidity, bending rigidity, surface area, and bulk acyl chain order within the bilayer (Pernpeintner et al., 2017; Urban et al., 2018; Doroudgar et al., 2021; Morstein et al., 2021). When combined with a protein in the lipid bilayer, some lipid-protein dynamics are shown to occur; Namely an increase in lipid packing, causing a decrease in Brownian motion through the membrane and amplifying the bilayer effects of the azo*-*lipid conformational change. Some site-specific changes were observed within a non-mechanosensitive transmembrane protein as it adapted itself to the change in bilayer fluidity, however, the system was not designed to trigger protein function (Doroudgar et al., 2021).

Applying FTIR spectroscopy allows insight into the molecular band changes that occur upon photoswitching, facilitating a new understanding of how the lipid bilayer plays its role in the opening of mechanosensitive proteins. We build nanodiscs containing the photolipid, which allow us to constrain the bilayer and stop the dissipation of lateral forces upon isomerization (Denisov et al., 2005; Mors et al., 2013; Viegas et al., 2016; Pardo et al., 2019). The nanodiscs enable the measurement of bulk samples, preserving the bilayer system.

In this work, we evaluate the photosensitive membrane as a trigger for mechanosensitive gating. We measure the change in lateral pressure achievable in an AzoPC monolayer when switching and deem it sufficient to trigger the open, conducting state of MscL using a Langmuir-Blodgett (LB) trough. MscL, when reconstituted into photoswitching AzoPC:DPhPC bilayers in an electrophysiological approach, is shown to be functional upon photoisomerization of the AzoPC, achieving a conducting sub-state but not the fully open state. Experimental and DFT modelled IR vibrational data are taken to characterize an AzoPC bilayer photoisomerization event. These vibrational bands are then used to follow the photoactive biomembrane switching in nanodiscs containing MscL, as changes in the protein due to the membrane switch give rise to differences in the amide I spectral range. These amide changes are interpreted as the result of the force generated by the photoswitching lipid bilayer, in such a way that an at-least partially conducting state is achieved. We envisage future experiments that can incorporate nanodiscs to perform FTIR spectroscopy on single bilayer systems, such as Surface Enhanced InfraRed Spectroscopy (Zaitseva et al., 2010; Ataka et al., 2013).

## Materials and Methods

The lipids 1-stearoyl-2-[(E)-4-(4-((4-butylphenyl)diazenyl)phenyl)butanoyl]-sn-glycero-3-phospho-choline (AzoPC), 1,2-distearoyl-sn-glycero-3-phosphocholine (DSPC), 1-palmitoyl-2-oleoyl-glycero-3-phosphocholine (POPC), and 1,2-diphytanoyl-sn-glycero-3-phosphocholine (DPhPC) were purchased from Avanti Polar Lipids (Alabama, United States) in chloroform with >99% nominal purity. For the LB trough and for all water-based solutions, deionized water was used.

The lateral pressure exerted during switching of a monolayer of AzoPC was quantified using a commercial LB trough, a Kibron MicroTroughX (Kibron, Finland). The trough was filled with pure water, onto which AzoPC in chloroform was deposited, forming a surface monolayer. In order to investigate the specific ability of AzoPC to generate a lateral tension change by light-induced isomerization, 100% pure AzoPC was used. The lipid distribution was initially 200 Å^2^/lipid, which corresponds to the gas phase. Barriers on either side of the trough were brought together to compress the lipid monolayer into a condensed phase, up to 30 mN/m. A dynamometer measures lateral pressure via a metal rod in contact with the surface. A continuous measurement of the surface tension was recorded and the lateral pressure exerted by the monolayer is calculated using:

$$\pi =\gamma_{0}-\gamma$$

where π is lateral pressure, γ_0_ is the surface tension of the pure subphase and γ is the surface tension of the monolayer at the air/water interface. It is noted that an increase in tension of the membrane, such as to open MscL, corresponds to a decrease in lateral pressure.

Switching of the monolayer was triggered by two light sources; A blue LED array emitting at 455 nm illuminated the surface with a power density of 10 mW/cm^2^. The second was a UV lamp emitting at 365 nm with a power density of 30 μW/cm^2^ at the surface. Illumination times of the lipid monolayer was 10 min each for one cycle.

MscL activity tests were performed on an Orbit Mini (Nanion Technologies GmbH, Germany) electrophysiological setup with a recording chip (MECA4) (100 μm; Ionera Technologies, Germany). The recording chip cavities were first filled with PBS buffer. DPhPC and AzoPC in a ratio of 80:20 and total concentration of 10 mg/ml were first mixed in chloroform, dried and then resuspended in Hexane. The lipid solution was illuminated with UV light prior to the application to generate the cis state of the AzoPC lipids. The lipid mixture was then painted as a planar lipid bilayer on the recording wells. DPhPC is a standard lipid used for freestanding lipid bilayers: its branched structure guarantees structural stability to black lipid membranes. 2 μl of MscL reconstituted in nanodiscs dissolved in PBS buffer were added and incubated for 1 h, a constant voltage of 20 mV was applied to promote protein insertion. Measurements were conducted at 25°C at a constant voltage of +20 mV, with cycles of 2 min continuous UV illumination followed by 2 min continuous blue light illumination. Recordings were filtered with −3 dB cutoff of 600 Hz and analyzed using Clampfit 10.4 software (Molecular Devices, United States).

IR spectra of AzoPC in the *trans* and *cis* states were calculated by applying density-functional theory (DFT) using the BP86/6-311+G* level of theory. Molecular designs of both configurations were done with Avogadro software and geometry was pre-optimized using the UFF force field. Calculations were run with Gaussian16 (Gaussian Inc., United States) in two steps, one for geometry optimization and a second one for normal mode (NM) analysis. NM frequencies were corrected using a linear scaling equation (Palafox, 2018). The experimental frequencies of 11 normal modes with a well-known assignment were plotted versus the values observed in the simulation. Scaling equation was obtained by linear fit and then applied to correct all other NMs. Line broadening was simulated using a set of Gaussian distributions centered at the NMs characteristic frequencies, all with the sigma value of 7 cm^−1^. More details on the DFT calculations, the data analysis and the band assignment are available in the Supplementary Material.

The expression and purification protocol has been adapted from Martinac (Martinac et al., 2010). For each protein expression, pET11a containing the MSCL gene with a 10xHis tag at the C-terminus was freshly transformed into the *E. coli* strain BL21-CodonPlus^™^ (DE3)-RP (Stratagene) and grown overnight at 37°C on BHI-agar plates (Brain Heart Infusion Agar) supplemented with 200 μg/ml ampicillin. The cells were harvested from the plates and transferred to dYT (double yeast extract-tryptone) medium with 200 μg/ml ampicillin for the main culture and incubated at 37°C. At OD_600_ 0.8–1.0, the cultures were inducted with 0.2 mM IPTG (isopropyl β-D-1-thiogalactopyranoside) for 3 h. Cells were pelleted for 15 min at 12,300 ×g at 4°C by centrifugation and resuspended in PBS buffer at pH 7.2 followed by disruption in a cell disruptor (Constant Systems, Model TS, 1.1 kW) at a pressure of 1.7 kbar. The cell lysate was continuously treated with DNAase and protease inhibitors (20 μg/ml PMSF, 10 μg/ml benzamidine) and additionally with a protease inhibitor tablet (cOmplete ™, Roche), then centrifuged for 30 min at 15,970 ×g at 4°C. The supernatant was collected and centrifuged for additional 3.5 h at 186,000 ×g at 4°C. The pellet was homogenized with PBS buffer and solubilized overnight at 4°C with 2% DDM (n-dodecyl-β-D-maltoside). The next day the solution was centrifuged for 1 h at 183,000 ×g at 4°C and the supernatant was loaded onto a Ni-NTA column (Protino, Macherey-Nagel) in an ÄKTA avant system. Washing steps were as follows: PBS buffer, followed by 20 mM imidazole in PBS pH 7.2 and then 60 mM imidazole in PBS pH 7.2. Elution was executed with the gradual increase of imidazole to 1 M in PBS buffer pH 7.2. The eluted protein was pooled and concentrated with a concentrator (Amicon Ultra-15, 50,000 MWCO, Merck). Additional purification was done using size exclusion chromatography (Superdex 200 Increase 10/300 GL Healthcare GE) on the ÄKTA avant system.

The His-tagged scaffold protein MSP1D1 was generated according to previously published protocols (Bayburt et al., 2002; Denisov et al., 2004). The plasmid pET28a-MSP1D1 was transformed in *E. coli* strain BL21-CodonPlus^™^ (DE3)-RP. The main culture was grown in TB-medium while supplemented with 50 μg/ml kanamycin and induced with 1 mM IPTG in the late logarithmic phase. The cells were centrifuged and resuspended in 300 mM NaCl, 40 mM Tris-HCl, pH 8.0, and 1% Triton X-100. Several cycles of ultrasonication were performed to disrupt the cells and the lysate was clarified using centrifugation. The solution was filtered (0.45 μm pore size) and subsequently purified using a Ni-NTA column for affinity chromatography, followed by size exclusion chromatography (Superdex 200 Increase 10/300 GL, Sigma-Aldrich) with a buffer exchange to 100 mM NaCl, 20 mM Tris-HCl, pH 7.4, 0.5 M EDTA. The eluted fractions were analyzed using SDS-PAGE.

For the reconstitution of MscL in nanodiscs, a mixture of AzoPC and POPC (50:50 w) was used. POPC, although not native for *E. coli* plasma membrane, has been proven to be well tolerated by the protein and to preserve the ion channel function (Moe and Blount, 2005; Bavi et al., 2017b). POPC and AzoPC for the reconstitution of nanodiscs were dissolved separately in 100 mM sodium cholate, 20 mM Tris and 100 mM NaCl for a final 50 mM stock solution. Nanodisc assembly for the sample measured in the FT-IR was done at a ratio 1:2:30:30 of MscL/MSP1D1/POPC/AzoPC and at a ratio of 1:2:60 of MscL/MSP1D1/POPC for the electrophysiology experiments. The mixture was incubated for 4 h at 4°C before biobeads (SM-2 resin, Bio-Rad) were added and rotated overnight in an overhead shaker at 4°C to remove detergent. The next day, the biobeads were removed and the sample was purified by size exclusion chromatography (Superdex 200 Increase 10/300 GL, Healthcare GE) on ÄKTA avant with 0.5 ml/min in PBS buffer at 25°C. Absorbance was monitored constantly at 280 nm, 365 and 455 nm.

FTIR spectroscopy has been carried out on Vertex 70 spectrometer (Bruker, Germany) as follows: 2,000 coadditions were recorded and averaged for sufficient signal-to-noise ratio. All graphs shown here report averages of 7 spectra, recorded with 4 cm^−1^ resolution. Spectra have been recorded in two configurations: For attenuated total reflection (ATR) measurements, about 10 µl of sample are gently dried on a silicon internal reflection element with a dry air flux and a circular motion. For the transmission experiment, the same drying procedure is done on a BaF_2_ window, then the sample is sandwiched with another window sealed with a greased spacer.

The rehydration in D_2_O of one of the nanodisc samples has been obtained by addition of a D_2_O/glycerol mixture in droplets around the sample deposited in ATR configuration and finally sealed with an O-ring and a window from the top.

## Results

**FIGURE 1:**
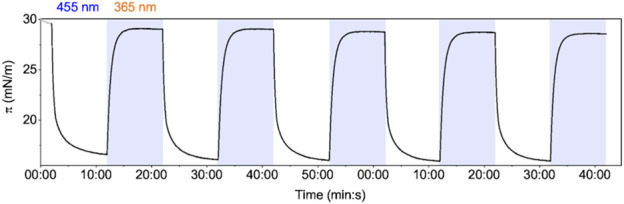
Light switching of the AzoPC monolayer: LB trough lateral pressure changes induced by intermittent light switching (blue/UV) on a monolayer of AzoPC at the air-water interface.

We first set out to quantify the tension induced by isomerization of an AzoPC monolayer formed in a LB trough. We also tested the reproducibility and consistency in light switching of AzoPC monolayer at the air/water interface. The AzoPC molecules, deposited on the water, were converted to the *cis* state by UV illumination prior to compression of up to 30 mN/m. In the *cis* state, the monolayer is set in the high pressure/low tension state of the membrane. When blue light is turned on, isomerization from *cis* to *trans* isomeric state of the azobenzene moiety is induced leading to pressure drop (Figure 1). Switching is fully reversible by UV illumination and reproducible over the recorded time of 100 min. The tension exerted by the AzoPC phase under the applied conditions is determined to be 13 mN/m. A plot of the switching starting from the *trans* state is shown in Supplementary Figure S1. The exponential fit to the kinetics of contraction and expansion of the monolayer are reported in Supplementary Figure S2. We have also observed a dependency of the tension change by the starting lateral pressure (Supplementary Figure S3).

As the previous set of experiments demonstrated that light-induced isomerization of AzoPC generates changes in lateral tension of a monolayer and it was shown that a tension of about 10 mN/m is sufficient to fully open MscL (Sukharev et al., 1999; Kung et al., 2010; Nomura et al., 2012), we set out to reconstitute MscL in a painted lipid membrane and probe the functional opening of MscL by electrophysiology. The black lipid membrane was composed of a 20:80 mixture of AzoPC and DPhPC. AzoPC was fully converted to the *cis* isomer by illumination with UV light while the black lipid membrane was formed. Prizl et al. observed that membrane permeability due to photoswitching *cis* to *trans* and *vice versa* may increase due to the formation of pores and some other defects in the bilayer (Pritzl et al., 2020). Thus, we have performed electrophysiological recordings as a control on the mixed AzoPC/DPhPC bilayer prior to insertion of MscL. We have observed that switching from *cis* to *trans* and *vice versa* under an applied voltage of +20 mV does not create any defects in the bilayer that may lead to leak currents (Supplementary Figure S4). The same study suggests (Pritzl et al., 2020) that mixing AzoPC with other lipids or compounds may reduce such phenomena which is hereby confirmed.

**FIGURE 2:**
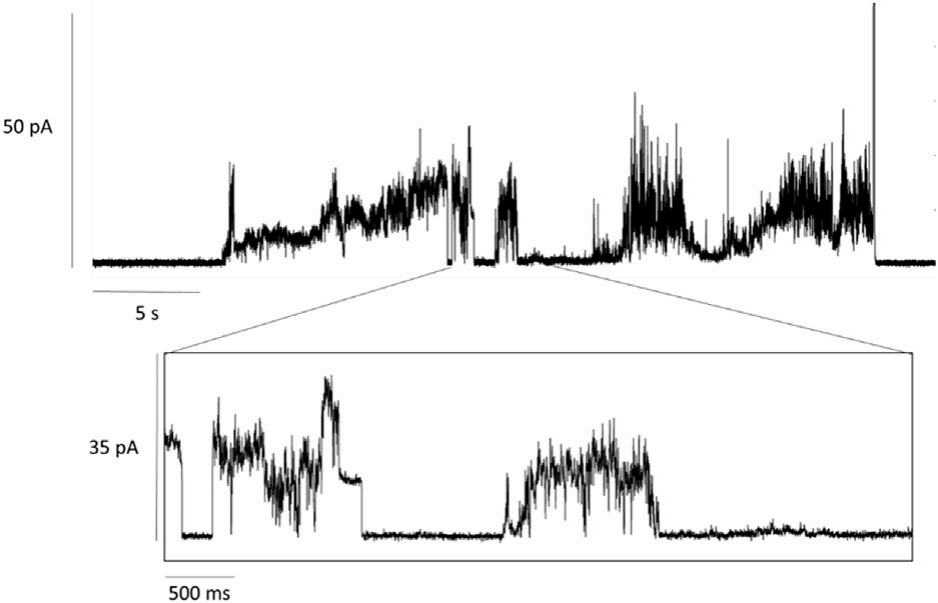
Current traces of MscL activation. MscL reconstituted into a mixed lipid bilayer of 20% AzoPC and 80% DPhPC. Activation of MscL was achieved by blue-light illumination (λ = 455 nm) of *cis*-AzoPC which lead to spontaneous openings of the channel after a delay time of about 5 s. The voltage across the bilayer was set to +20 mV.

MscL embedded in lipid nanodiscs was added to the bath solution and insertion into the lipid bilayer was driven by applying a transmembrane voltage of +20 mV. As *cis*-AzoPC generates low tension in the bilayer, we assume the MscL is in the closed state. Upon illumination of the bilayer with blue light, AzoPC isomerized to the trans state resulting in a change in the bilayer’s lateral pressure. Apparently, mechanical stress within the bilayer was established leading to spontaneous opening of MscL. The electrochemical gradient generated by the transmembrane voltage drives ions across MscL which is recorded as spontaneous currents (Figure 2). A threshold level of current was defined at 16 pA, above which we expect to see the first conducting substate of MscL. (Sukharev et al., 1999). This corresponds to an estimated conductance of around 0.8 nS (Supplementary Figure S6A). A significant channel event is shown in the enlarged window below the trace in Figure 2, where the current increases in a stepwise fashion. The experimental results were essentially reproduced on a different sample and day (Supplementary Figures S5, S6B for the analysis). Our electrophysiological experiments demonstrate not only successful insertion of MscL into the lipid bilayer, but also that functionality can be remotely triggered by light-induced isomerization of AzoPC.

**FIGURE 3:**
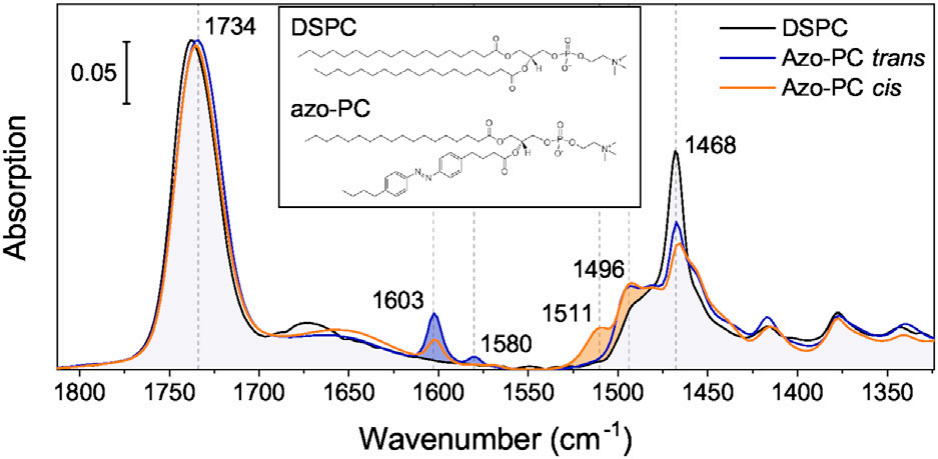
IR spectra of AzoPC compared to DSPC. IR absorption spectra of DSPC (black) and AzoPC in the *trans* (blue) and *cis* (orange) state are overlaid to contrast their vibrational differences. Blue-filled are the modes arising due to the *azobenzene*-modified lipid tail mostly characteristic of the *trans* state, orange-filled mostly of the *cis* state. The inset contains a chemical sketch of both lipid structures. Spectra over the full mid-IR (MIR) range are available in Supplementary Figure S7.

Now that we have demonstrated the activation via photo-isomerization of AzoPC, we studied the light-induced structural changes of MscL by FTIR spectroscopy. We identified vibrational marker bands of AzoPC by contrasting with ATR FT-IR spectrum of the related lipid DSPC (Figure 3). Overlapping bands reflect the similarities in structure, whereas bands that are only present in the AzoPC spectrum are indicative of the vibrational modes of azobenzene in either *trans* or *cis* isomeric states. Marker bands are labelled in Figure 3 by their respective frequencies and the vibrational assignment was performed on the basis of DFT calculations (Table 1). Assignment across the full mid-IR range is available in Supplementary Table S2, as well as the plots comparing measured and calculated spectra (Supplementary Figures S8–S10).

**TABLE 1:** Marker bands of AzoPC and their vibrational assignment.

1,735	C=O Stretching (sum of two Bands: 1,742 anhydrous esters, 1,730 hydrated esters)
1,603	Ring breathing mode of *trans* AzoPC
1,580	Ring breathing mode of *trans* AzoPC
1,511	N=N stretching of *cis* AzoPC
1,496	Ring breathing mode of *cis* AzoPC
1,468	CH_2_ scissoring mode

**FIGURE 4:**
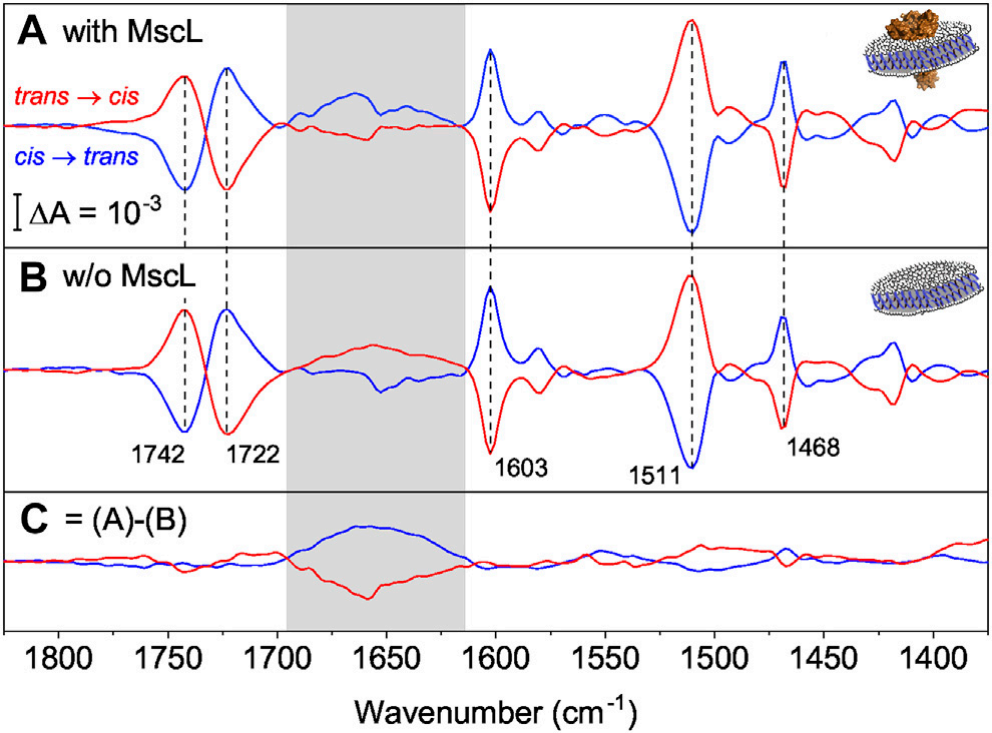
**(A)** FTIR difference spectra of nanodiscs supplemented with AzoPC in the presence of MscL (blue for the *cis* to *trans* switching, red for the *trans* to *cis*). Vertical dashed lines indicate marker bands for the isomerization of AzoPC. **(B)** Same as **(A)** but in the absence of MscL. **(C)** Double-difference spectra that reflect the response of MscL by subtracting difference spectra in the absence **(B)** from spectra in the presence of MscL **(A)**. The box refers to the amide I range which is indicative for changes in the C=O peptide bond, here of MscL (Insets). A diagram of a nanodisc with **(A)** and without **(B)** MscL reconstituted. The ion channel is in orange, lipids are in white and the scaffold proteins in blue.

The strong band centered at around 1,735 cm^−1^ represents the accumulative absorption of the two esters located in the glycerol backbone with the peak of the anhydrous C=O located at 1,742 cm^−1^, and the hydrated C=O at around 1,730 cm^−1^ (Blume et al., 1988). The two components become evident in the difference spectrum (Figure 4). Several marker bands are identified by contrasting the IR bands of *trans* and *cis* AzoPC. The *trans* state of AzoPC is identifiable by sharp peaks at 1,603 and 1,580 cm^−1^ which are assigned to azobenzene ring breathing modes (Table 1). The *cis* state is characterized by the bands at 1,511 and 1,496 cm^−1^, which are the different ring breathing modes that arise after the isomerization of the azobenzene. These assignments are in agreement with those from other FTIR studies on azobenzene (Duarte et al., 2014).

The vibrational assignment facilitates the interpretation of the light-induced FTIR difference spectra. For these experiments, MscL was reconstituted in nanodiscs (AzoPC:POPC, 50:50 w) to provide a physical constraint to the lateral pressure exerted by isomerization of AzoPC. We recorded FTIR difference spectra (Figure 4A) for both switching directions, cis to trans (blue trace) and trans to *cis* (red trace). Marker bands of AzoPC (Figure 4A) are clearly evident from the spectra of the nanodiscs and used to qualitatively gauge the tension of the biomembrane. Reversibility and reproducibility of switching is confirmed for at least seven cycles (Supplementary Figures S11–S13). Data presented is the average of seven cycles of photoswitching. The almost exact mirror image of the light-induced difference spectra recorded after *cis* to *trans* and *trans* to *cis* isomerization is another indication of the high reproducibility and reversibility of the experiments.

As a control sample, the same preparation of nanodiscs were used, but omitting MscL. The recorded light-induced difference spectra exhibited very similar absorption changes (Figure 4B), in particular for the marker bands of AzoPC (dashed vertical lines). In both samples, the nanodiscs with MscL and the control, the transition from the *cis* to the *trans* state show a positive band for the ring breathing mode, characteristic of the trans state, at 1,603 cm^−1^, and a negative band for the mode of the *cis* state at 1,511 cm^−1^, confirming our assignment. Inverted signs for these bands are then observed for the opposite transition. The two components of the C=O stretching band (Blume et al., 1988) are clearly separated in the difference spectrum. The sigmoidal shape reflects a change in hydration, with the *trans* state more hydrated than the *cis* state. From linearly polarized difference spectra (Supplementary Figure S14) we were able to link the change in hydration to a reorientation of the carbonyl groups upon azobezene isomerization, where in the *trans* state there is an increase in the population of carbonyls exposed to water, with respect to the *cis* state. The CH_2_ scissoring mode of the alkyl chains also reorients by the light switch and it shows a clear band at 1,468 cm^−1^. The supporting polarized spectra are reported and commented in Supplementary Figure S14. Close inspection of the amide I range (gray area) revealed band features distinctively different to those in the presence of MscL (Figure 4A). The absorption differences in the amide I range (Figure 4B) in the absence of MscL indicate small tension-induced structural changes in the scaffold protein MSP1D1.

To exclusively resolve the structural changes of MscL, double-differences have been calculated where the light-induced difference spectra in the absence of MscL (Figure 4B) have been subtracted from those in the presence of MscL (Figure 4A). The resulting spectra (Figure 4C) show the strongest band features in the amide I region (highlighted as gray box in Figure 4). Maxima and minima, respectively, are located at around 1,660 cm^−1^ which is characteristic to changes of α-helical structure, which is the dominant secondary structural element in the transmembrane part of MscL. Similar experiments in which H_2_O was replaced by D_2_O showed the same vibrational changes in the amide I range (Supplementary Figure S15). As the amide I band is hardly affected by H/D exchange, the isotopic replacement experiment excludes the contribution of vibrational bands due to water (here the scissoring mode of H-O-H in the range of 1,640 cm^−1^). As another control, light-induced difference spectra of pure AzoPC (Supplementary Figure S11) do not show this broad band in the amide I region; on the contrary, they show a broad absorption band with the opposite sign to the one we attribute to MscL, positive for the *trans* to *cis* switching and negative *vice versa*. Therefore, if there is a contribution from the AzoPC spectrum, this is being hidden by a larger MscL amide I band.

In summary, we clearly demonstrate here structural changes of α-helices take place in MscL which are induced by light activation of AzoPC. We refrain at this stage to interpret other spectral features which arise upon switching. Rather, we conclude that the spectral changes induced by MscL are related to the protein-lipid interactions in the nanodiscs. This is confirmed by testing the system function using the electrophysiological set-up, where changes in the photoactive membrane were sufficient to induce conductance in the protein.

## Discussion

We show the reproducibility and the reversibility of the photoswitching of AzoPC both in the monolayer and in the bilayer for at least seven cycles. The LB trough experiment on the monolayer shows a double exponential transition between the *cis* and the *trans* state in both directions, confirming previous findings (Pritzl et al., 2020). Since the kinetics are strongly dependent on the power density of the light, the time constants are setup-specific and cannot be generalized. The *cis* state is confirmed as having a higher steric hindrance, here measured as area/molecule, and therefore corresponding to a higher lateral pressure. To induce tension, the lateral pressure must be decreased. This is achieved by switching the lipids to the *trans* state by blue-light illumination. The magnitude of the pressure difference that can be induced depends on the starting lateral pressure; for higher starting lateral pressures a larger difference can be achieved. The pressure jump that we measured in the planar monolayer (13 mN/m) cannot be compared quantitatively to other lipidic systems, such as the liposomes or the nanodiscs; it is a good indication, however, of the system’s ability to achieve the threshold tension in order to trigger the protein.

MscL is a non-binary channel, meaning the channel can be found not just in its closed and open conducting state, but with multiple conducting substates as the surface tension is increased until it reaches the threshold for the open state. The group of states are as follows: C↔S1↔S2↔S3↔O, where C is the closed state, S1-3 are the semi-conducting states and O is the open state (Sukharev, 1999; Sukharev et al., 1999). The state observed in our electrophysiological experiment is comparable to the semi-conducting state S1, corresponding to a signal amplitude of 16 pA at an applied voltage of 20 mV. The conductance measured at this substate is 0.8 nS.

We have chosen nanodiscs to not only as a mimic of a biomembrane but also to provide lateral constraint to the pressure exerted by switching of AzoPC (Denisov et al., 2005; Schachter et al., 2020). We then recorded the effects of light switching on MscL by FTIR spectroscopy. The assigned marker bands can be used to determine the state of the lipids in any system that contains them. The light-induced switching of the lipids changes tension within the lipid nanodisc which impacts the structure of MscL. As the amide I band is indicative of changes in the protein backbone and the frequency of the largest change is characteristic for α-helix, we infer that the transmembrane part of MscL is affected. The reversibility of these changes on MscL can be interpreted as a consistent rearrangement of the α-helices of MscL upon change in lateral pressure. We can affirm that as a consequence of UV or blue light illumination, MscL assumes two distinct stable conformations which are spectroscopically distinguishable.

Our interpretation of the spectral features, in combination with the measured conductance of 0.8 nS from our electrophysiological study, assert that the forces transferred from a photoswitching lipid bilayer to MscL are not sufficient to fully open MscL but induce semi-conduction in this mechanosensor. However, the measured conductance is not insignificant. Sukharev et al. (1999) estimated that the pore radius of the S1 substate is 0.8 nm. Such a radius would be more than sufficient to allow the efflux of small molecules when incorporated into a photopharmacological delivery system. It is also of note that the starting pressure of the lipid leaflet will affect its tension (Supplementary Figure S3). The black lipid membrane used for the current trace recordings does not have a strong physical constraint and can therefore restore its properties *via* reorganization and exchange with the boundary lipids in the annulus surrounding the recording well. It can therefore be speculated that the tension profile differs to that of the monolayer in the LB trough experiment. By inserting the photo-active membrane and the mechanosensor into nanodiscs, we add a constraint in the form of the scaffold protein, thereby increasing the starting tension of the bilayer. This may lead to a larger force communicated to the protein, and higher conductance than that measured in the electrophysiological experiment.

In conclusion, this work presented a photopharmacological approach to remotely activate mechanosensitive channels by light. We imagine that our results will open a route towards time-resolved studies of mechanosensation by applying pulsed laser sources. Thereby, kinetic information is retrieved on the temporal sequence of structural changes in mechanosensitive channels. Time-resolved crystallographic techniques can track these structural changes at utmost temporal and spatial resolution (Skopintsev et al., 2020; Mous et al., 2022), once the mechanosensing protein is co-crystalized with AzoPC. Finally, these experiments will derive an atomic picture of the lipid/protein coupling and resolve the gating mechanism of these channels. A long-term goal will be to use this insight to foster an understanding of the molecular mechanisms of hearing and pain, preparing new targets to restore the former and remedy the latter*.*

## Data Availability

The original contributions presented in the study are included in the article/Supplementary Material, further inquiries can be directed to the corresponding author.
