# Supplementary material for: Photoactivation of a Mechanosensitive Channel
Source: Front Mol Biosci. 2022 Jun 28;9:905306. doi: 10.3389/fmolb.2022.905306 (PMC9273776; doi:10.3389/fmolb.2022.905306)
Supplement: Supplementary file 1 [file DataSheet1.docx]

Supplementary Material

# Light-induced tension increase in the photo-lipid monolayer: switching direction, initial pressure dependence and time constants.

With the perspective of using AzoPC as an actuator of MscL channel gating, our interest was focused on setting the lipids in the *cis* state and monitoring the change happening upon blue light illumination, since it is the *cis* to *trans* transition that would generate tension in the membrane. In **Figure S1** we show also that, if starting from the same lateral pressure, 30 mN/m, but with the monolayer prepared in the *trans* state and therefore switching it in the opposite direction, *trans* to *cis*, the amplitude of pressure change is of 48 mN/m. This shows that the pressure jump depends on the starting pressure. We tried light-switching from different starting pressure conditions: the results are shown in **Figure S2**. In **Figure S3**, kinetics of contraction and expansion of the monolayer have been fitted with the following sum of exponentials: $y = y0+ A1*\exp\left( -\frac{x-x0}{t1} \right)+ A2*\exp\left( -\frac{x-x0}{t2} \right)$

The fits converged (χ^2^ tolerance set at 1E-9) and the values are reported in **Table ST1**. A similar analysis was done by Pritzl *et al.* (Pritzl et al., 2020), finding different time constants. The reported values are in fact valid for the setup used and cannot be generalized, since the time constants strongly depend on the energy density of the light sources used.


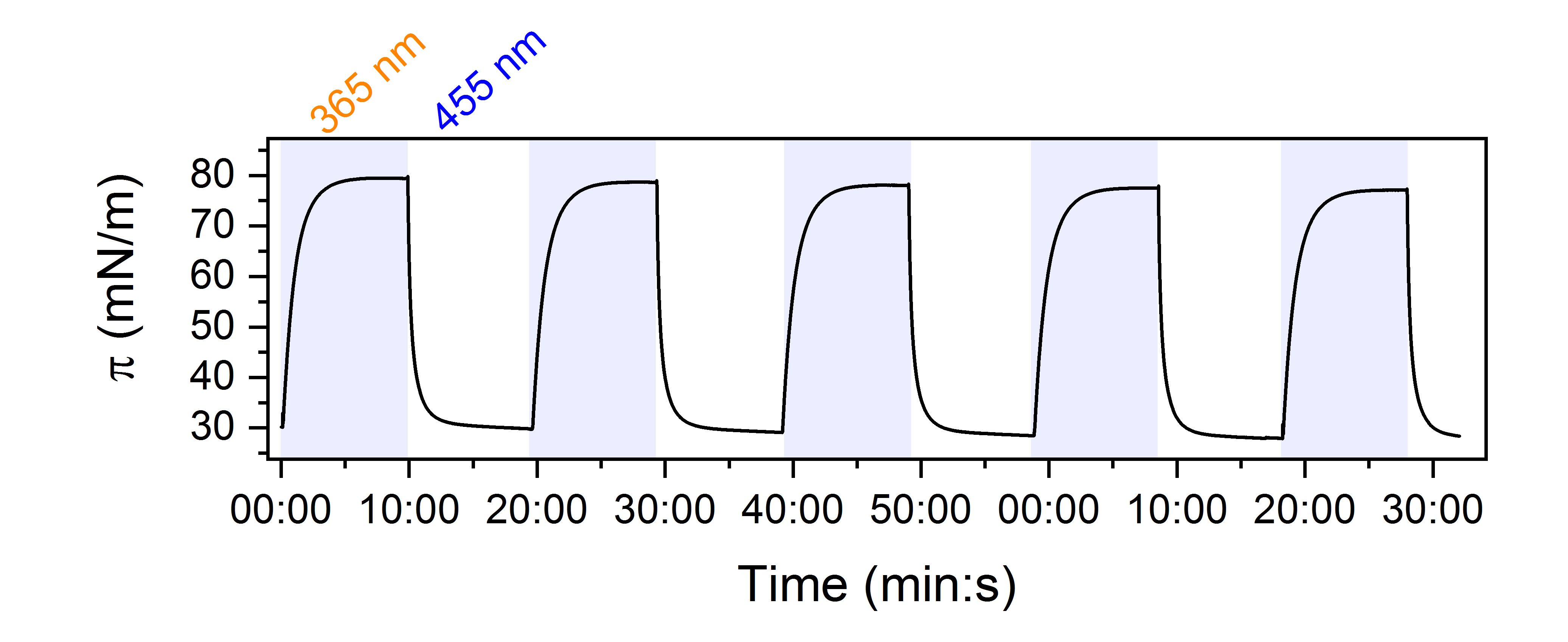


Figure S1. Langmuir trough lateral pressure changes induced with intermittent light switching (blue and UV light) on a monolayer of AzoPC at the air-water interface, starting from 30 mN/m in the *trans* state.


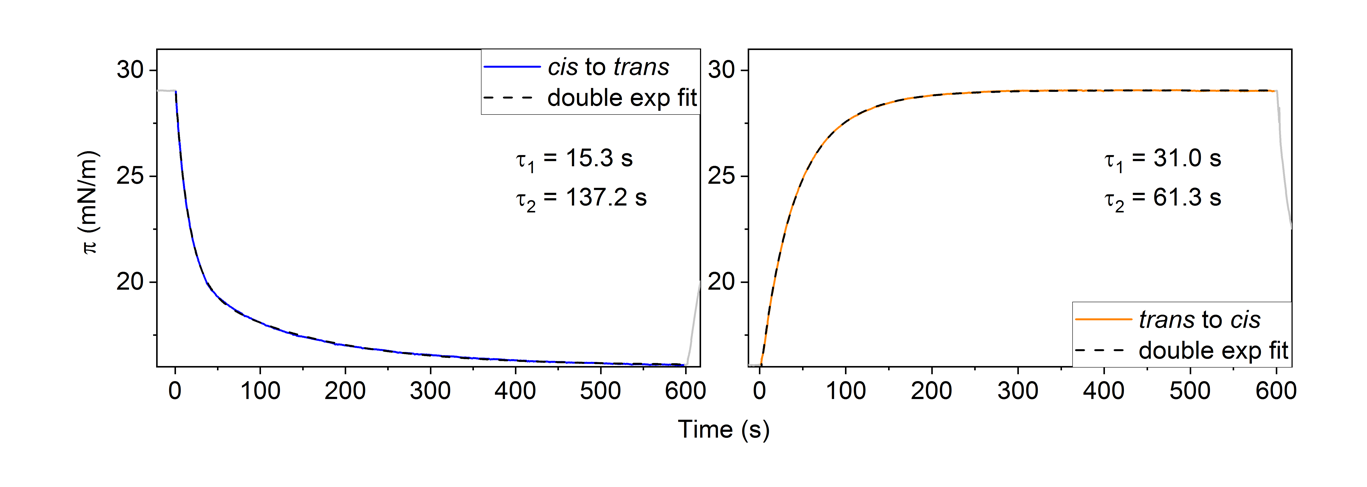


Figure S2. Double exponential fitting of the kinetics of the contraction (left) and expansion (right) of the AzoPC monolayer. The parameters of the fit are reported in Table ST1.

**Table ST1.** Values for the double exponential fit of the contraction (A) and the expansion (B) of the AzoPC monolayer shown in **Figure S2**.

| **A** | Value | Standard Error |  |  |  | **B** | Value | Standard Error |
| --- | --- | --- | --- | --- | --- | --- | --- | --- |
| y0 | 16.06331 | 0.00145 |  |  |  | y0 | 28.59738 | 3.56E-04 |
| x0 | -3.58144 | 179079.9 |  |  |  | x0 | 1.74465 | 105766.1 |
| A1 | 11.47467 | 134353.3 |  |  |  | A1 | -6.56752 | 24140.74 |
| t1 | 15.29462 | 0.02846 |  |  |  | t1 | 28.77378 | 0.11173 |
| A2 | 4.27211 | 5574.29 |  |  |  | A2 | -5.93019 | 10335.63 |
| t2 | 137.2459 | 0.30426 |  |  |  | t2 | 60.68455 | 0.19842 |


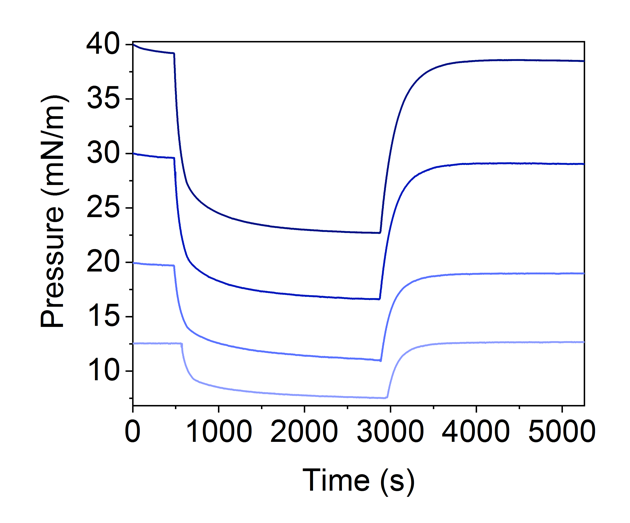


Figure S3. The intensity of the tension that it is generated in the monolayer of AzoPC depends on the starting lateral pressure. The higher the initial lateral pressure is, the larger tension is developed upon light switching.

# Electrophysiology


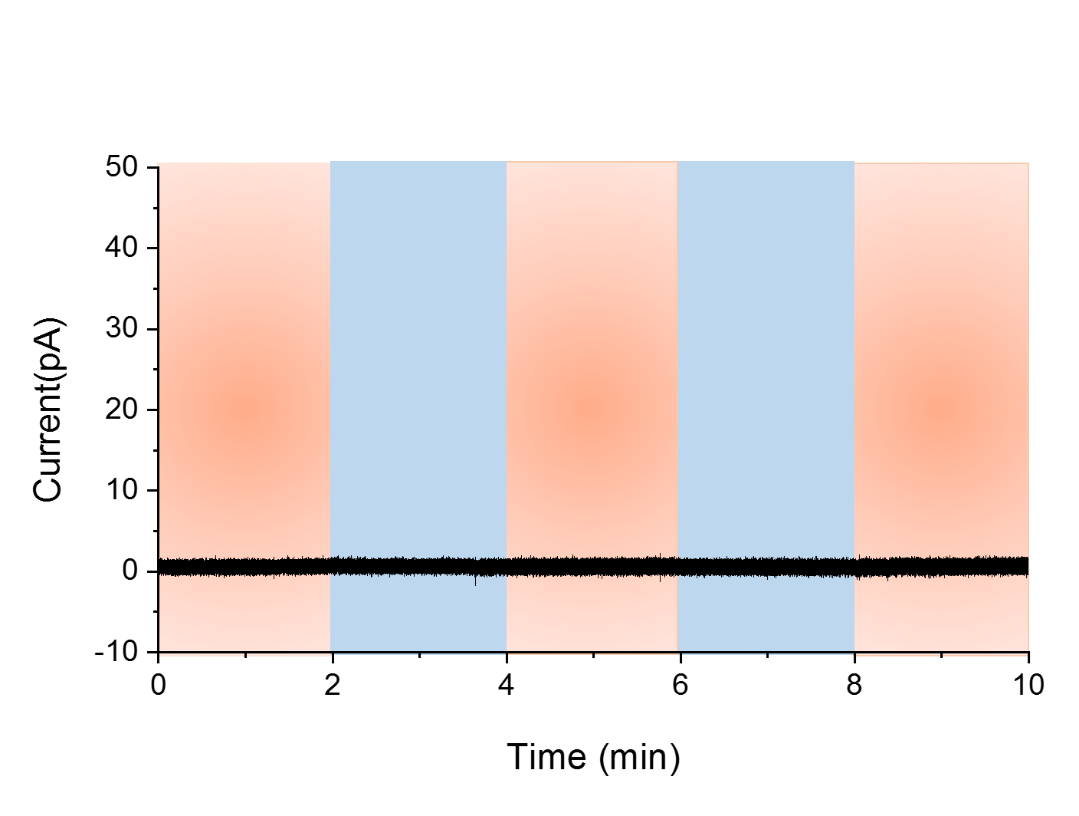


Figure S4. Negative control of the photoactive lipid bilayer. A continuous recording is shown of the AzoPC:DPhPC bilayer, without protein. The bilayer is illuminated initially with UV light for 2 minutes (365 nm, orange region) followed by blue light for 2 minutes (450 nm, blue region). The illumination steps are repeated for a 10 min period, while a constant tension of +20 mV is applied. No conductance was observed over the full timescale, meaning that the bilayer remained intact for the duration of the measurement and photoswitching alone is not sufficient to see a current flow across the membrane.


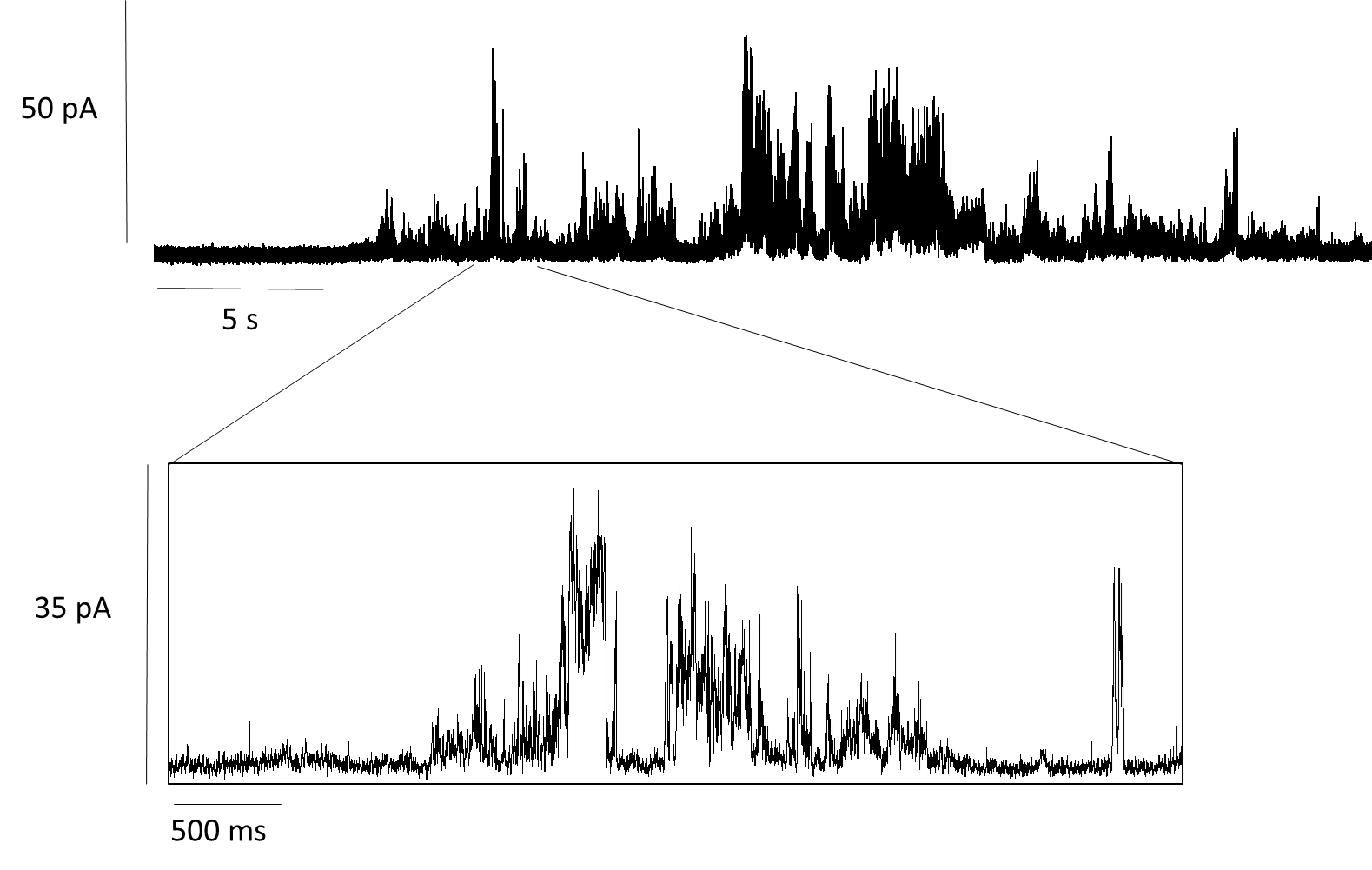


Figure S5. Current Trace of MscL in a conducting state after photoswitching: as a supplement to the trace presented in Figure(2), +20mV applied voltage and blue light illumination. The statistical analysis of the activity recorded in this current trace is reported in Figure S6B.

B

A


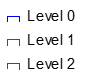


Figure S6. Amplitude histogram of all events of the recording region. The current traces of Figure(2) (A) and Figure S5 (B) were processed by defining threshold levels of current by which to distinguish the substates of the protein. Level 0 was set as the baseline or zero current, corresponding to a closed state. Level 1 was set to a nominal current level, where some current passed through the bilayer after switching but before the channel would reach a characterized substate. All histograms were fitted with a Gaussian function with the Levenberg-Marquardt method and with 1 term. (A) amplitudes of 2.6 pA for level 0, 5.8 pA for level 1 and 15.9 pA for level 2. Calculated conductance of level 2 is 1.25nS which agrees with the literature value of the subconducting state S1.Similar (B) Mean amplitudes of 2.5 pA for level 0, 6.6 pA for level 1 and 13.1 pA for level 2 and 23.85 pA for level 3. Calculated conductance of level 2 is 1.52 nS which agrees with the literature value of the subconducting state S1 (Sukharev, 1999, Sukharev et al., 1999). In figure (B) level 3 amplitude agrees with possible activation of 2 MscL channels in the lipid bilayer.

# Assignment of AzoPC

The assignment of the AzoPC IR absorption was done by a comparison with DSPC, combined with the DFT analysis on the MIR frequency range, from 4000 cm^-1^ to 800 cm^-1^. We report here the experimental spectra over the whole range (**Figure S4**), a direct comparison between the measured spectra of AzoPC in *trans* and *cis* state and their corresponding DFT calculated spectra over the whole MIR range (**Figure S5**) and a close up for the range of interest (**Figure S6**). The details of the DFT data analysis are further discussed in the **SI section 2.1.**

Although the DFT calculated spectra cannot reproduce in detail the features of the measured spectra, the relevant marker bands of AzoPC can be distinguished. Even if shifted of up to 10 wavenumbers, the CH_2_ and CH_3_ stretching vibrations of the aliphatic chains are well recognizable in the range 3030-2800 cm^-1^ (**Figure S5**). The two components of the C=O band measured at 1734 cm^-1^ (*cfr.* **Table 1** in the main text) are indeed visible in the DFT spectra, with few wavenumbers difference between the *cis* and the *trans* state (**Figure S6**). It is relevant to notice that, while the two components of the experimental band are assigned to the hydrated and anhydrous populations of esters in the sample (*cfr.* **Table 1** in the main text), in the DFT calculation on a single molecule in vacuum, they are assigned separately to the *sn*-1 and the *sn*-2 aliphatic chains, respectively to the highest and to the lowest wavenumbers. The ring breathing mode at 1604 cm^-1^ matches the experimental value, with a much stronger attenuation in the *cis* state with respect to the *trans* than for the experimental intensity. *Vice versa*, the ring breathing mode characteristic of the *cis* state, measured at 1511 cm^-1^ and shifted few wavenumbers higher in the simulation, is present in both states’ spectra, but with a higher intensity in *cis*, confirming our assignment. The calculated band at 1488 cm^-1^ for the *trans* state, at 1492 cm^-1^ in the *cis* state, is the sum the other ring breathing mode measured at 1496 cm^-1^ (*cfr.* **Table 1** in the main text) with the CH_2_ scissoring deformation measured at 1468 cm^-1^.


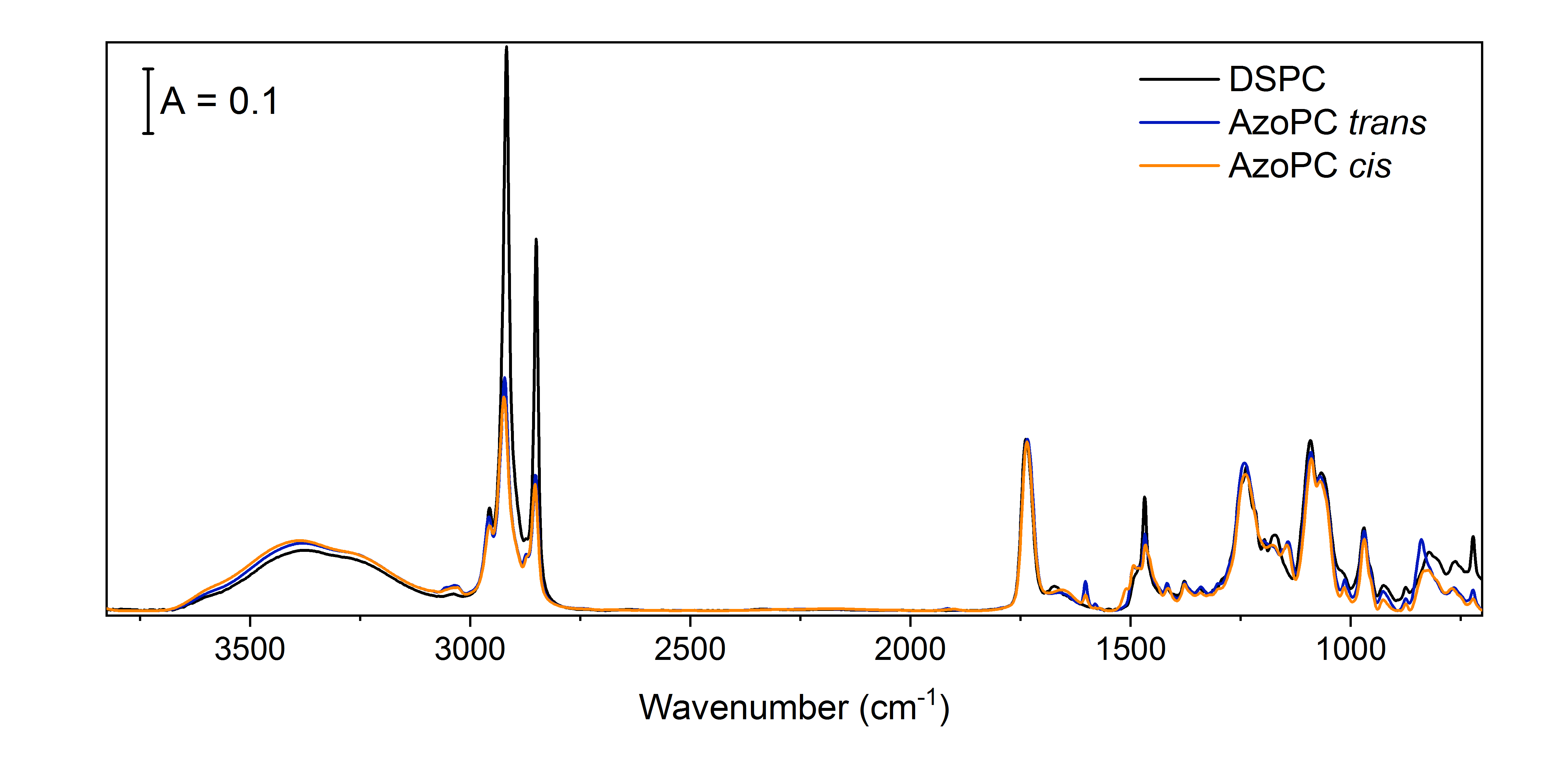


Figure S7. Attenuated total reflection (ATR) absorption spectrum of AzoPC in the two isomerization states, *trans* in blue and *cis* in orange, compared to DSPC (chemical structures of the lipids are in the inset of Figure 3 in the main text), over the MIR range.


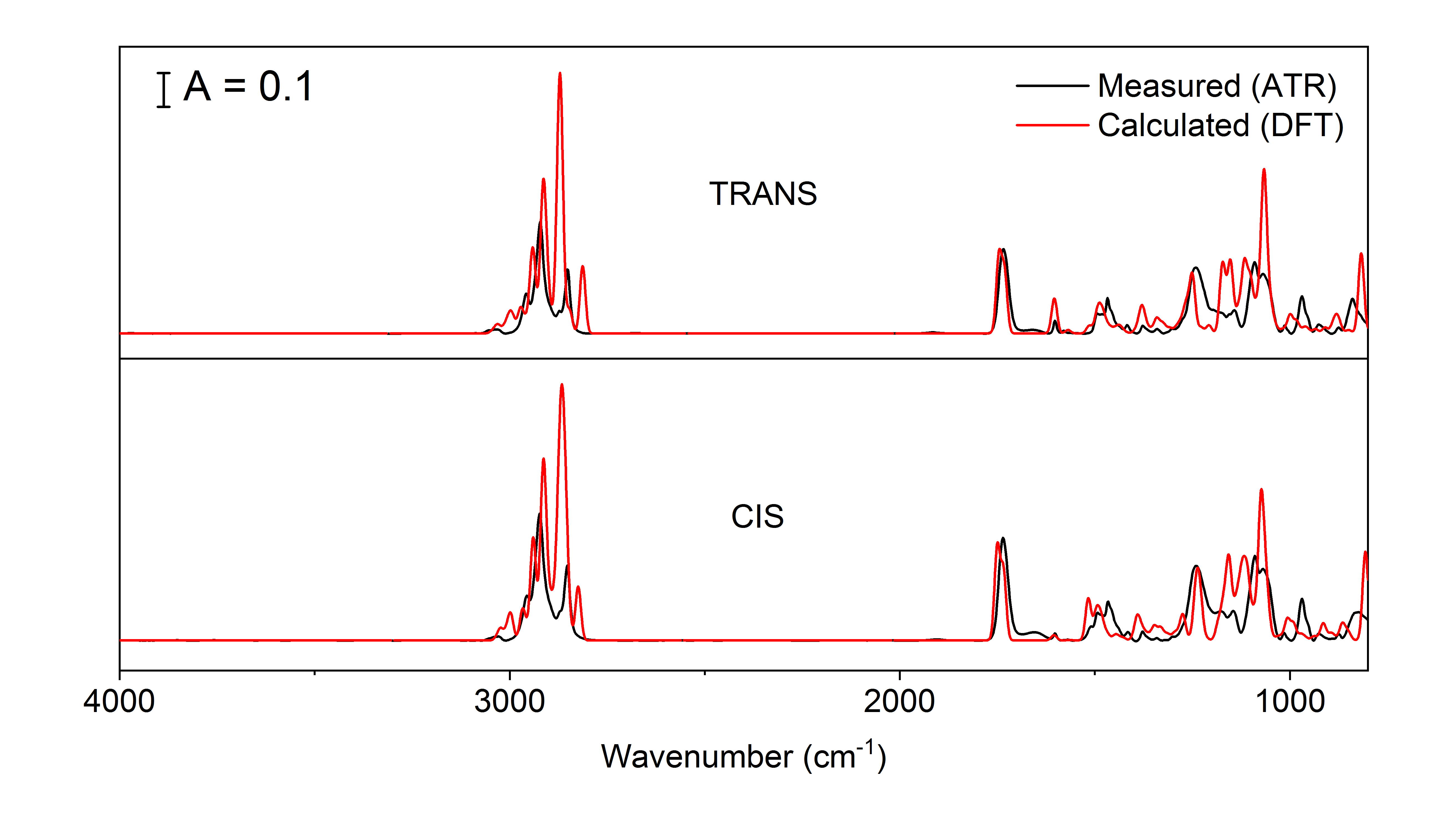


Figure S8. Comparison between ATR measured spectra and DFT calculated spectra over the full measured MIR range. DFT data are normalized to the measured ones at the C=O 1734 cm^-1^ band.


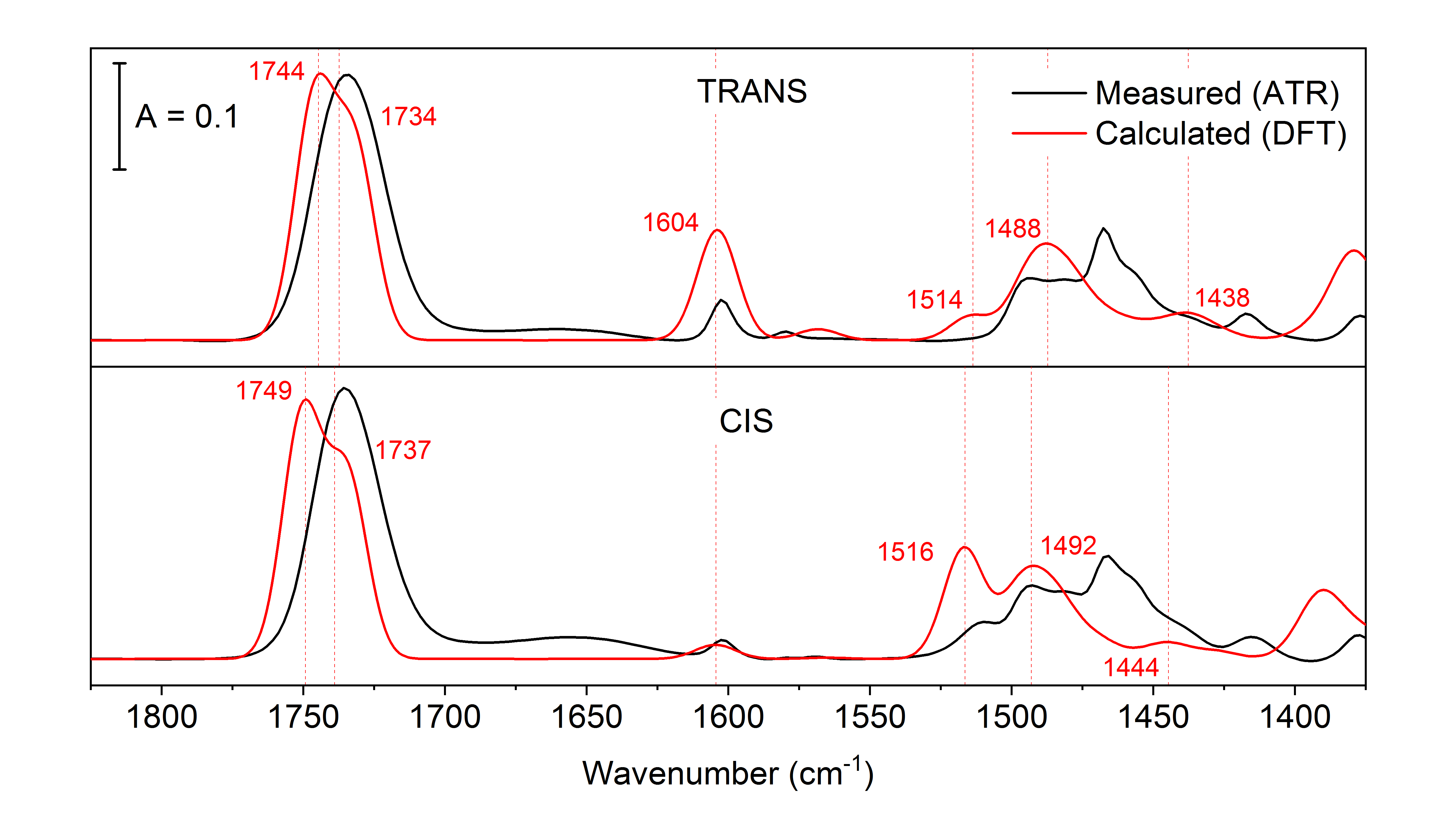


Figure S9. Detail of the *cis* AzoPC (top) and *trans* AzoPC (bottom) ATR and DFT vibrational spectra compared in the range of interest. AzoPC marker bands and their frequencies are highlighted and discussed in the text.

## DFT scaling factors and assignment of AzoPC marker bands

Density-functional theory (DFT) calculations are known to overestimate the wavenumbers of normal vibrational modes, mainly due to the limitations of theoretical models. The accuracy of calculations can be improved by choosing more complex basis sets and by including anharmonic effects but this inevitably results in longer calculation times. Particularly, when addressing big molecules (>100 atoms), a compromise has to be found between accuracy and affordable calculation times. In order to account for the limitations of theoretical models, there exist for most of them, pre-calculated correcting factors (National Institute of Standards and Technology (NIST)). It is however custom in research, to calculate these from direct comparison of theoretical predictions with experimental data (Palafox, 2018). Such a correction also partially accounts for frequency shifts introduced by the molecular environment, which are often neglected during the DFT modeling. In our system, we use a linear equation to correct the theoretical frequencies obtained from the BP86/6-311+G* basis set from the simulation of a single AzoPC molecule in vacuum.

Band assignment was first performed in the experimental spectra using information available from literature.(Mantsch and McElhaney, 1991, Blume, 1996, Tamm and Tatulian, 1997) The identified vibrational modes were then localized within the list of calculated normal modes (**Table ST2**). Experimental frequency values were plotted versus the theoretical ones (**Figure S7**) and data were fitted using a linear model. The obtained linear scaling equation was then applied to correct the complete list of normal vibrational wavenumbers.

It is often the case that vibrations of the same functional group can be observed in different normal modes close to each other in the theoretical calculation. Because of their proximity, the same cannot be distinguished in the experimental spectra and appear overlapped as a single band. Whenever this situation was found, experimental bands were assigned to the strongest of all normal vibrational modes containing the assigned vibration. This was the case for the stretching and deformation vibrations of CH_2_ and CH_3_ groups, which would appear at different frequencies depending on whether they belonged to the choline, aliphatic or azo tails. Experimental bands were assigned in this case to those of the aliphatic tail, which were the ones with the strongest intensity.

.
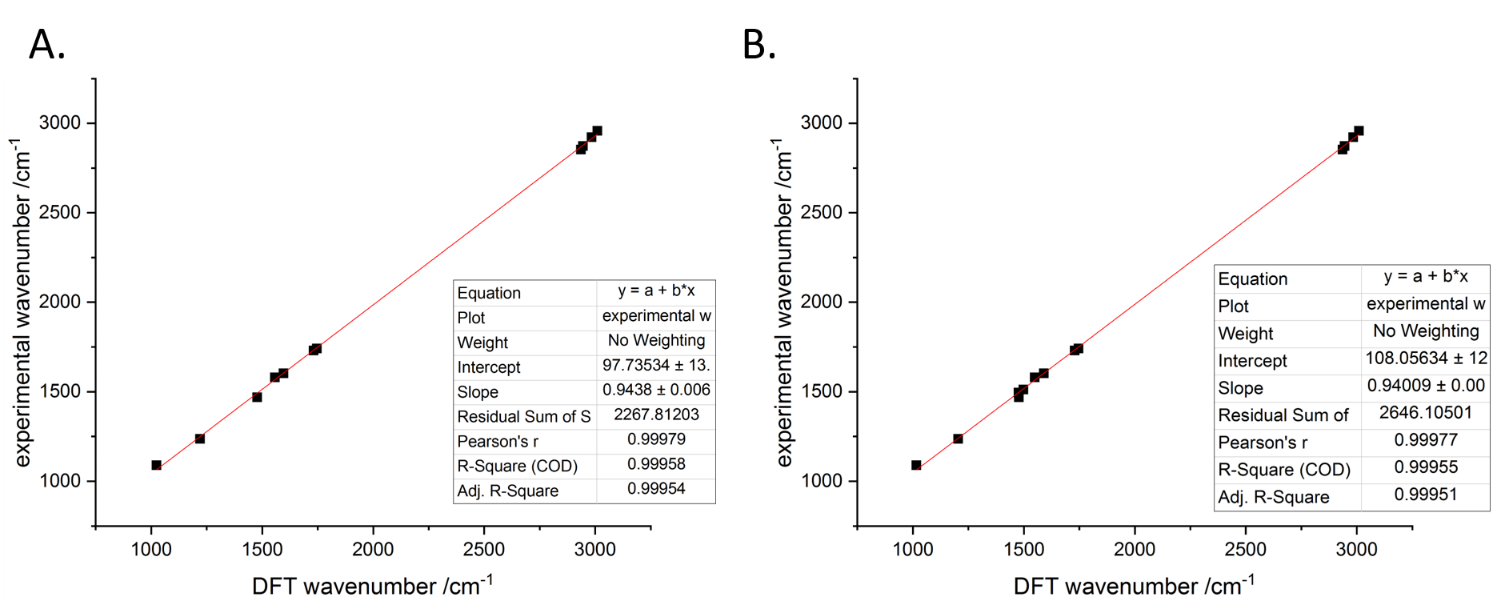


Figure S10. Scaling equation from linear fit of experimental data versus theoretical prediction. A) *trans* configuration, B) *cis* configuration. The data points correspond to the frequencies assigned from literature, summed up in Table ST2, and used for the linear fit to later correct all other absorption frequencies calculated from the DFT.

**Table ST2.** Band assignment and reference peaks used for calculation of linear scaling equation. Definitions: str. stretching, def. deformation, sym. symmetric, asym. asymmetric

**Trans isomer**

| **DFT** | **Experimental** | **Assignment** |
| --- | --- | --- |
| /cm^-1^ | /cm^-1^ |  |
| 1024.2 | 1090 | PO_2_ sym. str. |
| 1220.2 | 1237 | PO_2_ asym. str. |
| 1477.2 | 1468 | CH_2_ def. |
| 1557.7 | 1580 | Benzene ring breathing (b2) |
| 1595.7 | 1603 | Benzene ring breathing (a1) |
| 1731.2 | 1730 | C=O str. (azo branch) |
| 1746.5 | 1742 | C=O str. (aliphatic branch) |
| 2936.2 | 2852 | CH_2_ sym. str. |
| 2945.5 | 2873 | CH_3_ sym. |
| 2983.9 | 2922 | CH_2_ asym. str. |
| 3009.8 | 2957 | CH_3_ asym. str. |

**Cis isomer**

| **DFT** | **Experimental** | **Assignment** |
| --- | --- | --- |
| **/cm-1** | **/cm-1** |  |
| 1016.2 | 1090 | PO_2_ sym. str. |
| 1204.8 | 1237 | PO_2_ asym. str. |
| 1476.7 | 1496 | Benzene ring def. |
| 1477.2 | 1468 | CH_2_ def. |
| 1498.1 | 1512 | Azo str. + Benzene ring def. |
| 1549 | 1580 | Benzene ring breathing (b2) |
| 1590.9 | 1603 | Benzene ring breathing (a1) |
| 1730 | 1730 | C=O str. (azo branch) |
| 1746.9 | 1742 | C=O str. (aliphatic branch) |
| 2936.1 | 2852 | CH_2_ sym. str. |
| 2945.5 | 2873 | CH_3_ sym. |
| 2983.7 | 2922 | CH_2_ asym. str. |
| 3009.8 | 2957 | CH_3_ asym. str. |

# MIR difference spectra of pure AzoPC switching

The switching of pure AzoPC was observed in the full MIR range from a chloroform-dried sample on ATR. We report here on the reversibility and reproducibility of the switching over seven cycles, but no decrease of signal has been observed for over 50 cycles.


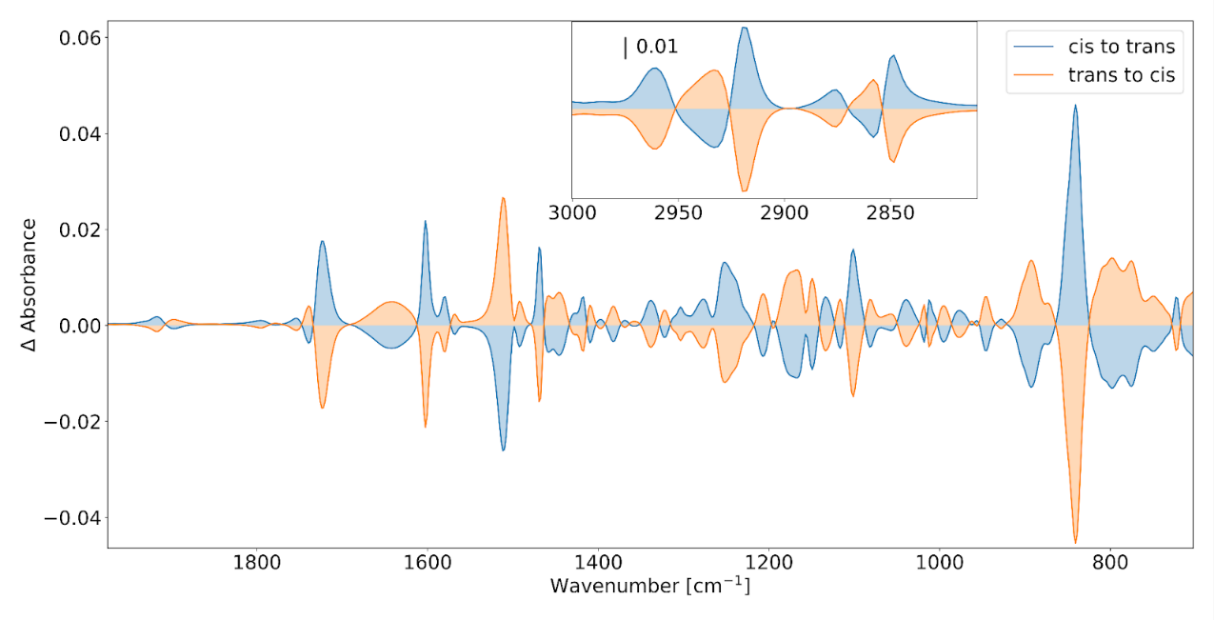


Figure S11. Reversibility of the switching in AzoPC. The difference spectra of the light-switching of pure AzoPC in the two directions (from *cis* to *trans* and from *trans* to *cis*) are mirror images, proving the reversibility of the light switching of AzoPC.


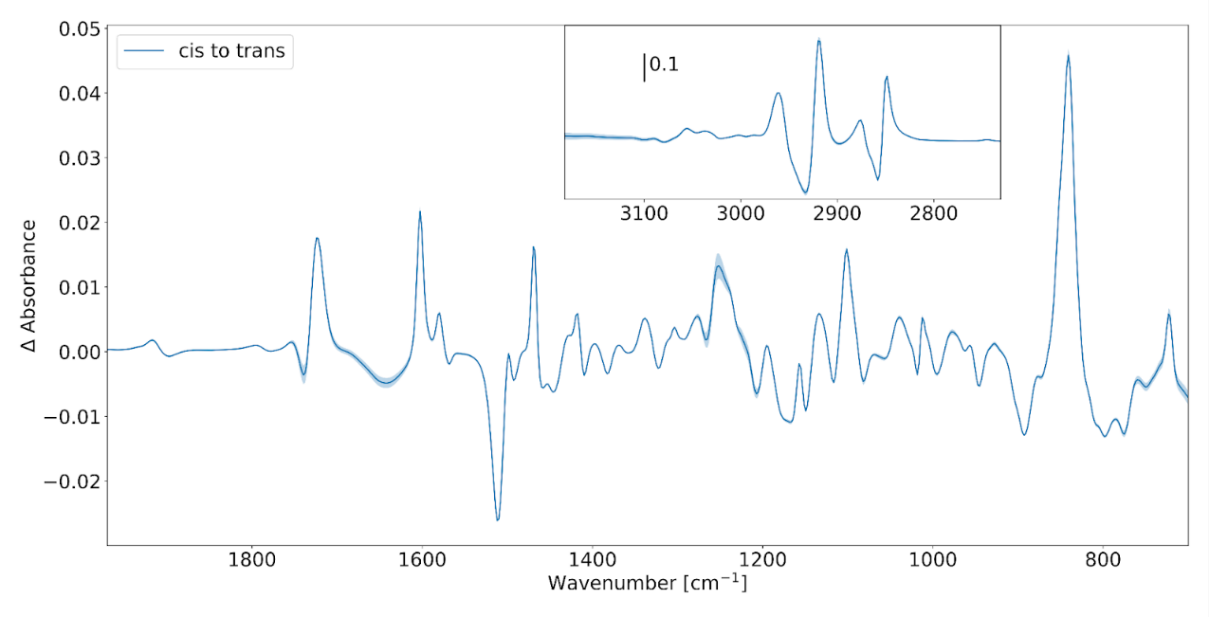


Figure S12. Reproducibility of the switching in AzoPC. The variance over seven switching cycles is shown as a shadowed area around the curve. The switching can be considered highly reproducible.

# Decrease of amide I signal in the nanodiscs

When MscL is reconstituted in a nanodisc containing AzoPC, seven cycles become a limit, differently from the pure AzoPC systems. The specific amide I signal visible at around 1655 cm^-1^ decreases with the number of cycles of light switching of the lipids. We can imagine that the activity and movement of the protein inside of the nanodisc induces some rearrangements to reach a new equilibrium, therefore diminishing the potential pull on the protein.





**Figure S13.** Full overnight measurement of light-switching MscL in nanodiscs (going from blue to red over switching cycles). The signal reported in the upper panel of Figure 4 in the main text is a subset of this. In particular only the first seven spectra of these (starting from blue) were averaged to obtain the spectrum in the upper panel of Figure 4. Here, in the inset, we quantified the relative difference in intensity between the maximum and minimum points characteristic of the specific amide I signal observed. The frequencies at which the intensities were taken are 1662 and 1652 cm^-1^, highlighted on the spectra with the dashed gray line. The difference in intensity decreases exponentially, as indicated by the fit in red.

# Polarized ATR-FTIR difference spectra

For the polarized measurement of dry AzoPC on ATR, we defined the XY plane as the plane of the lipidic membrane, and the Z axis as the normal to it. The spectra are collected in two polarizations, parallel and perpendicular to the plane of incidence to the internal reflection element of the ATR optics. They are then converted into XYZ coordinates as previously explained in detail (Lórenz-Fonfría et al., 2009). By drying the lipids from chloroform we create a stack of bilayers. The sample is well ordered, as reflected by the high dichroism of its difference spectrum. The axis of lipid molecules and their aliphatic chains are mostly oriented along the Z axis, while the CH_2_ bonds, being almost perpendicular to that axis, lay in the XY plane. **Figure S13** is the linearly polarized difference spectrum of AzoPC. In the region of 2800 cm^-1^ to 3000 cm^-1^, a significant component of the ν(CH_2_) and ν(CH_3_) stretching vibrations shift from the Z axis (indicated by negative peaks in the red trace) into the XY plane (positive peaks, black trace) as the lipids are switched from *cis* to *trans*. Similarly, the scissoring mode whose dipole moment also lays in the XY plane in the *trans* state, appears as a positive band in the XY polarized spectrum for the *cis* to *trans* transition. The inset shows the ν(C=O) bands which arise upon switching the azobenzene. The strong dichroism indicates a reorientation of the C=O bond from a rather Z-aligned position in the *cis* state to a more XY-planar one in the *trans* state. A shift from higher wavenumbers (the large negative band at 1737 cm^-1^ along the Z axis) to lower (positive band at 1724 cm^-1^ in the XY plane) indicates that the ester group is moved from a less H-bonded environment to a more hydrated state after the photoswitch.





Figure S14. Polarized ATR-FTIR difference spectra upon isomerization of AzoPC from *cis* to *trans* (baseline corrected to remove features due to water). Inset: zoom in of the C=O stretching vibration.

# FT-IR spectra of MscL in nanodiscs rehydrated in D_2_O

Liquid water bending mode absorbs at 1650 cm^-1^. Changes in hydration can often be misinterpreted as amide I changes, due to the frequency overlap. In the double difference spectrum of MscL in nanodiscs we observe a broad band centered in 1655 cm^-1^ that we identified as an amide I change from MscL (*cfr*. **Figure 4C** in the main text). This band is reversible following the light switching, and reproducible, which allows us to correlate it to changes happening upon sample illumination. In order to exclude completely hydration changes, we measured the same sample of MscL reconstituted in nanodiscs rehydrated in D_2_O.


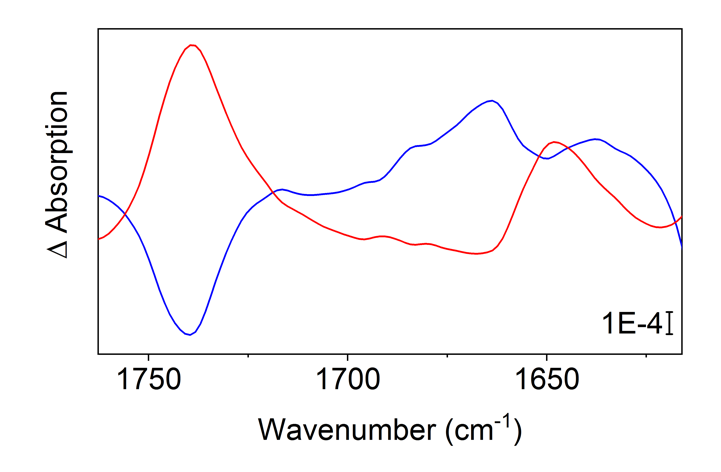


Figure S15. Difference spectrum of MscL in nanodiscs rehydrated in D_2_O. In red the *trans* to *cis* transition, in blue the *cis* to *trans*. The large band at 1735 cm^-1^ of the C=O stretching is included as reference for the eye. The broad absorption centered at 1655 cm^-1^ is visible also in the D_2_O rehydrated sample, therefore an assignment to a change in H_2_O hydration can be excluded.

A. Blume (1996). Properties of lipid vesicles: FT-IR spectroscopy and fluorescence probe studies. 1**,** 64-77.

V. A. Lórenz-Fonfría, M. Granell, X. León, G. Leblanc & E. Padrós (2009). In-Plane and Out-of-Plane Infrared Difference Spectroscopy Unravels Tilting of Helices and Structural Changes in a Membrane Protein upon Substrate Binding. *Journal of the American Chemical Society,* 131**,** 15094-15095.

H. H. Mantsch & R. N. Mcelhaney (1991). Phospholipid phase transitions in model and biological membranes as studied by infrared spectroscopy. *Chemistry and Physics of Lipids,* 57**,** 213-226.

National Institute of Standards and Technology (Nist). *Computational Chemistry Comparison and Benchmark Database (CCCBDB)* [Online]. Available: <https://cccbdb.nist.gov/vibscalejust.asp> [Accessed].

M. A. Palafox (2018). DFT computations on vibrational spectra: Scaling procedures to improve the wavenumbers. *Physical Sciences Reviews,* 3.

S. D. Pritzl, P. Urban, A. Prasselsperger, D. B. Konrad, J. A. Frank, D. Trauner & T. Lohmüller (2020). Photolipid Bilayer Permeability is Controlled by Transient Pore Formation. *Langmuir,* 36**,** 13509-13515.

S. Sukharev (1999). Mechanosensitive channels in bacteria as membrane tension reporters. *The FASEB Journal,* 13.

S. I. Sukharev, W. J. Sigurdson, C. Kung & F. Sachs (1999). Energetic and spatial parameters for gating of the bacterial large conductance mechanosensitive channel, MscL. *Journal of General Physiology,* 113**,** 525-539.

L. K. Tamm & S. A. Tatulian (1997). Infrared spectroscopy of proteins and peptides in lipid bilayers. 30**,** 365-429.
